# Analysis of the Transcriptome of *Erigeron breviscapus* Uncovers Putative Scutellarin and Chlorogenic Acids Biosynthetic Genes and Genetic Markers

**DOI:** 10.1371/journal.pone.0100357

**Published:** 2014-06-23

**Authors:** Ni-Hao Jiang, Guang-Hui Zhang, Jia-Jin Zhang, Li-Ping Shu, Wei Zhang, Guang-Qiang Long, Tao Liu, Zheng-Gui Meng, Jun-Wen Chen, Sheng-Chao Yang

**Affiliations:** 1 Yunnan Research Center on Good Agricultural Practice for Dominant Chinese Medicinal Materials, Yunnan Agricultural University, Kunming, Yunnan, People's Republic of China; 2 Kunming University of Science and Technology, Kunming, Yunnan, People's Republic of China; Chinese Academy of Medical Sciences, Peking Union Medical College, China

## Abstract

**Background:**

*Erigeron breviscapus* (Vant.) Hand-Mazz. is a famous medicinal plant. Scutellarin and chlorogenic acids are the primary active components in this herb. However, the mechanisms of biosynthesis and regulation for scutellarin and chlorogenic acids in *E. breviscapus* are considerably unknown. In addition, genomic information of this herb is also unavailable.

**Principal Findings:**

Using Illumina sequencing on GAIIx platform, a total of 64,605,972 raw sequencing reads were generated and assembled into 73,092 non-redundant unigenes. Among them, 44,855 unigenes (61.37%) were annotated in the public databases Nr, Swiss-Prot, KEGG, and COG. The transcripts encoding the known enzymes involved in flavonoids and in chlorogenic acids biosynthesis were discovered in the Illumina dataset. Three candidate cytochrome P450 genes were discovered which might encode flavone 6-hydroase converting apigenin to scutellarein. Furthermore, 4 unigenes encoding the homologues of maize P1 (R2R3-MYB transcription factors) were defined, which might regulate the biosynthesis of scutellarin. Additionally, a total of 11,077 simple sequence repeat (SSR) were identified from 9,255 unigenes. Of SSRs, tri-nucleotide motifs were the most abundant motif. Thirty-six primer pairs for SSRs were randomly selected for validation of the amplification and polymorphism. The result revealed that 34 (94.40%) primer pairs were successfully amplified and 19 (52.78%) primer pairs exhibited polymorphisms.

**Conclusion:**

Using next generation sequencing (NGS) technology, this study firstly provides abundant genomic data for *E. breviscapus*. The candidate genes involved in the biosynthesis and transcriptional regulation of scutellarin and chlorogenic acids were obtained in this study. Additionally, a plenty of genetic makers were generated by identification of SSRs, which is a powerful tool for molecular breeding and genetics applications in this herb.

## Introduction

*Erigeron breviscapus* (Vant.) Hand-Mazz. is a famous medicinal plant. The major active components of *E. breviscapus* are flavonoid glucuronides [1], mainly scutellarin, along with a small amount of apigenin 7-*O*-glucuronide and quercetin-3-*O*-glucuronide [2]. Scutellarin is a kind of flavonoid 7-*O*-glucuronide, which has neuroprotective effects and cytotoxicity protective action [3], [4], [5]. Flavonoid 7-*O*-glucuronides are commonly detected in plants within the Lamiales order, such as *Perilla frutescens*, *Antirrhinum majus*, and *Scutellaria baicalensis* [6], but the high content of scutellarin is only found in a few *Erigeron* species, including *E. breviscapus* and *E. multiradiatus* [7].

The aglycon of scutellarin, scutellarein, is a kind of flavone (5,6,7,4′-tetrahydroxyflavone). In plants, flavones are synthesized at a branch point of the anthocyanidin/proanthocyanidin pathway; however, flavanones are known as the direct precursor of flavones [8]. The biosynthesis of flavone and other flavonoids is well characterized [9], some genes encoding the enzymes involved in the biosynthesis of flavonoids have also have been cloned and identified from *E. breviscapus*, such as chalcone isomerase (CHI), chalcone synthase (CHS) and flavone synthase II (FSII) gene [10], [11], [12]. The 6-OH is the most important characteristic of scutellarin, suggested that there must be a flavonoid 6-hydroxylase (F6H) in *E. breviscapus*, which converts apigenin to scutellarein. Some F6Hs have been characterized for their activities of hydroxylation the 6-C in the A-ring of flavonoids [13], [14], [15], but F6H in *E. breviscapus* is still unclear.

**Figure 1:**
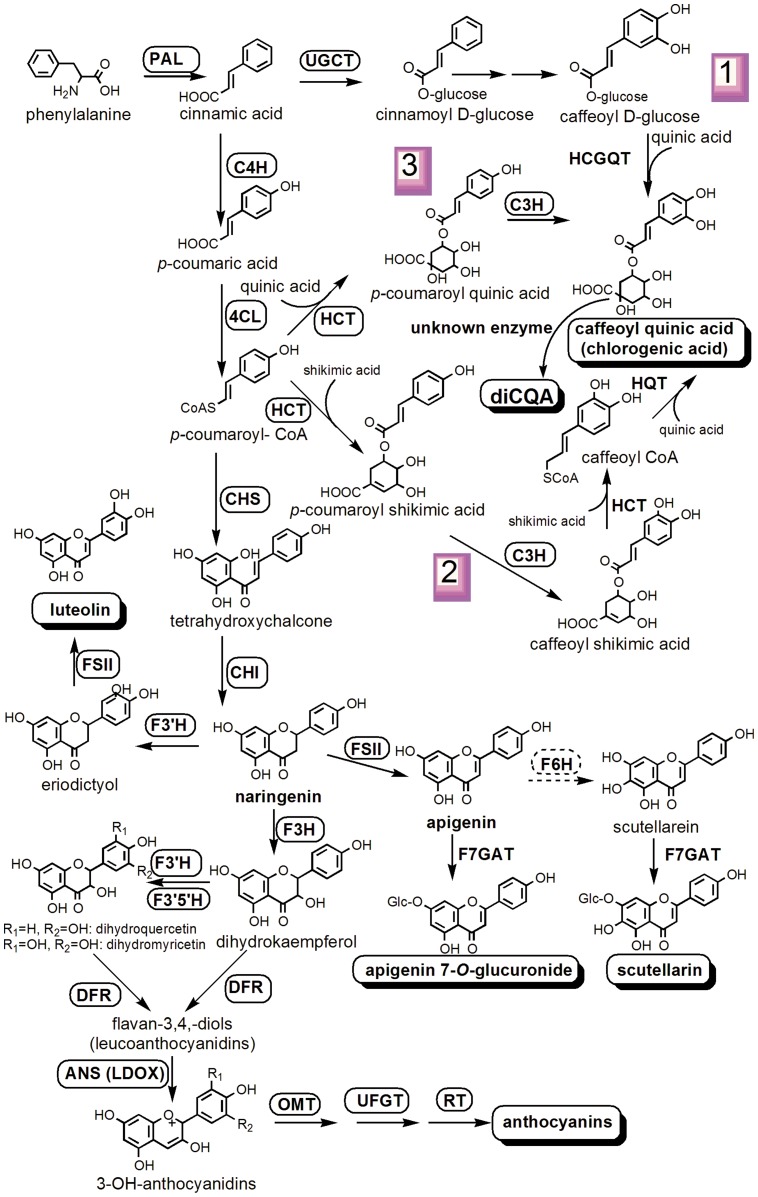
Putative pathway for scutellarin and chlorogenic acids biosynthesis in *E. breviscapus*. The major products previously found in *E. breviscapus* are surrounded by shaded boxes. Enzymes found in this study are boxed; new putative enzyme (flavone 6-hydroase, F6H) was surrounded by dashed line box. Enzymes involved in the pathways are: 4CL, 4-coumarate: CoA ligase; C3H, *p*-coumarate 3-hydroxylase; C4H, cinnamate 4-hydroxylase; CHI, chalcone isomerase; CHS, chalcone synthase, DFR, dihydroflavonol 4-reductase; F3′5′H flavonoid 3′5′ hydroxylase; F3′H, flavonoid 3′ hydroxylase; F3H, flavanone 3-hydroxylase  =  flavonol synthase (FLS); FSII, flavone synthase II; HCGQT, hydroxycinnamoyl D-glucose: quinate hydroxycinnamoyl transferase; HCT, hydroxycinnamoyl CoA shikimate/quinate hydroxycinnamoyl transferase; HQT, hydroxycinnamoyl CoA quinate hydroxycinnamoyl transferase; LDOX, leucoanthocyanidin dioxygenase  =  anthocyanidin synthase (ANS); OMT, *O*-methyltransferase; PAL, phenylalanine ammonia lyase; RT, rhamnosyl transferase; UFGT, UDPG-flavonoid glucosyl transferase; UGCT, UDP glucose: cinnamate glucosyl transferase; F7GAT, flavonoid 7-O-glucuronosyltransferase.

Chlorogenic acids (CGAs) are other kind active components of *E. breviscapus*, which have the same healthy functions as scutellarin [16]. CGAs act also as antioxidants in plants and protect against degenerative and age-related diseases in animals [17]. In *E. breviscapus* CAGs mainly include1,5-*O*-dicaffeoylquinic acid (1,5-diCQA), 3,4-diCQA, 3,5-diCQA, 4,5-diCQA, and erigoster B [18], [19], [20]. In plants, CQAs are important intermediates in lignin biosynthesis [21], and they are derived from phenylpropanoid as flavonoids do [22]. Three distinct pathways have been proposed for the synthesis of CGA, many enzymes involved in the biosynthesis of CGA have been identified [23]–[30], but the molecular mechanism for formation of di-caffeoylquinic acid from caffeoylquinic acid is still unclear. [24], [31] (Figure 1). Furthermore, scutellarin and CGAs are both derived from phenylpropanoid pathway, both of which share the same precursor cinnamic acid or *p*-coumaroyl-CoA (Figure 1). Therefore, *E. breviscapus* is an ideal material for elucidating the biosynthesis and regulation of flavone and CGAs.

*E. breviscapus* belongs to the family of the Asteraceae and is distributed in the southeastern region of China, mainly in Yunnan, Guizhou, Sichun, and Guangxi Provinces [32]. This herb is commonly used for treating various paralysis and its sequelae [16], and also for relieving exterior syndrome, dispelling the wind and dampness, and removing stagnancy of indigested food in folklore [33]. However, the wild resource of this herb is endangered due to overexploitation [34]. Our research group had accomplished the domestication and cultivation of *E. breviscapus*. Moreover, based on mass selection, we had bred two varieties with high yields and high quality. The content of scutellarin in the two varieties is more than 3.0% [35], which is at least 4 times higher than that in the wild plants [36]. Unfortunately, available genomic information on this herb is unavailable, especially the biosynthesis and regulation of scutellarin and chlorogenic acids is considerably unknown.

The objective of the present study is to analyze the transcriptome of *E. breviscapus* using Illumina sequencing on GAIIx platform, and to discover all candidate genes encoding enzymes and putative transcription factors involved in scutellarin and chlorogenic acids biosynthesis. Based on RNA-seq, lots of simple sequence repeats (SSR) markers could be found, this will facilitate marker-assisted breeding of this plant.

## Materials and Methods

### Ethics statement

No specific permits were required for the described field studies. No specific permissions were required for these locations and activities. The location is not privately-owned or protected in any way and the field studies did not involve endangered or protected species.

### Plant material

*E. breviscapus* cv. “Qianshan 1” was grown at the experimental fields of Honghe Qianshan Technology Co., Ltd in Lu-xi County, Yunnan province, southwest of China (24° 36′ 36″N, 103° 49′ 30″E, alt. 1710 m), which is a variety with high scutellarin content and selected from wild germplasm in Lu-xi County [35]. The mature leaves (Figure S1A) and flowers at full-blooming stage (Figure S1B) were harvested from one-year-old plants. All of the samples were immediately frozen in liquid nitrogen and stored at −80°C until use.

### RNA-Seq library construction and sequencing

Total RNA was extracted from young fresh leaves and different stage of flowers using RNeasy Plant Mini Kit (Qiagen) following by the RNA purification by RNeasy MiniElute Cleanup Kit (Qiagen), according to the manufacture's protocol. Equal amounts of RNA from each sample were mixed together for the subsequent steps of our experiments. For mRNA library construction and deep sequencing, at least 20 µg of total RNA samples were prepared by using the TruSeq RNA Sample Preparation Kit (Illumina) for Illumina sequencing on Genome Analyzer IIx platform at CapitalBio Corporation (Beijing, China). The high quality reads obtained in this study have been deposited in the NCBI SRA database (accession number: SRA111764).

### Illumina reads processing and assembly

Transcriptome *de novo* assembly was carried out using the short read assembly program: Trinity. A Perl program was written to remove low quality reads (reads with a base quality less than 20). Then the high quality reads were *de novo* assembled by using Trinity program [37], [38] at k-mer lengths of 25.

### Functional annotation and predicted CDS

Functional annotations were performed by sequence comparison with public databases. All unigenes were compared with the non-redundant protein database (Nr, http://www.ncbi.nlm.nih.gov/) and the SWISS-PROT database (http://www.expasy.ch/sprot) using BLSATX (E-value <1e^−5^) [39] and BLASTX (E-value <1e^−10^). Unigenes were also compared with the Clusters of Orthologous Groups of proteins database (COG) (http://www.ncbi.nlm.nih.gov/COG/) [40] and Kyoto Encyclopedia of Genes and Genomes database (KEGG, release 58; http://www.genome.jp/kegg) [41] using BLASTX with an E-value of less than 1e^−10^, and then a Perl script was used to retrieve KO (KEGG Orthology) information from BLASTX result and then established pathway associations between unigenes and database. InterPro domains [42] were annotated by InterProScan [43] Release 27.0 and functional assignments were mapped onto Gene Ontology (GO) (http://www.geneontology.org/) [44]. Then we made GO classification and drew GO tree using WEGO (http://wego.genomics.org.cn/cgibin/wego/index.pl) [45].

We predicted the CDS (Coding sequences) using blastx and ESTscan. we first performed BLASTX alignment (E-value<10^−5^) between unigenes and protein databases like Nr, Swiss-Prot, KEGG and COG. The best alignment results were used to determine the sequence direction of unigenes. Unigenes with sequences having matches in one database were not searched further. When a unigene would not align to any database, ESTScan was used to predict coding regions and determine sequence direction.

### EST-SSR detection and primer design

Using MISA tool [46] (http://pgrc.ipk-gatersleben.de/misa/), the potential SSR markers with motifs ranging from di- to hexa-nucleotides in size were detected among the 73,092 unigenes. The minimum of repeat units were set as follows: six for dinucleotides and five for tri-, tetra-, penta- and hexa-nucelotides. Primer pairs flanking each SSR loci were designed using the Primer3 program (http://primer3.ut.ee/).

### Survey of EST-SSR polymorphism

A total of 36 primer pairs (File S1) were synthesized and thirteen *E. breviscapus* accessions (File S2) were selected for polymorphism investigation with the EST-SSRs. Total DNA was isolated from *E. breviscapus* leaves using the CTAB method. PCR amplifications were conducted in a final volume of 20 µL containing 1 µL 2.5 mM dNTPs,1 µL *EasyTaq* DNA polymerase (Beijing TransGen Biotech Co., Ltd. China), 2 µL 10×*EasyTaq* buffer, 1 µL of each primer (10 µM), 13 µLddH_2_O and 1 µL template DNA (approx. 10 ng/µL). PCR was performed as follows: denaturation at 94°C for 2 min, followed by 35 cycles of 30 s at 94°C, 30 s at Tm (annealing temperature), 30 s at 72°C and a final step at 72°C for 5 min. The separation of alleles was performed on a 8% polyacrylamide gel. PCR products were mixed with an equal volume of loading buffer. The mixture was denatured at 95°C for 5 min before loading onto the gel.

### Genetic diversity and data analysis

The presence of each single band was coded as 1 and its absence as 0 in a data matrix. The genetic diversity and mean allele number were calculated using Popgene version 1.32 [47]. Scoring data from polymorphic loci were used to calculate the polymorphism information content (PIC) according to the formula of PIC  = 1–∑pi^2^ (pi is the frequency of i^th^ allele for each locus) [48].

By NTSYS pc 2.1 program [49], Jaccard's genetic similarity coefficients were calculated and dendrogram was constructed by the UPGMA (un-weighted pair group method with arithmetic mean) clustering method.

### Phylogenetic Analysis

Phylogenetic analysis based on the deduced amino acid sequences of CYPs and R2R3-MYB transcription factors from *E. breviscapus* and other plants. All of the deduced amino acid sequences were aligned with Clustal X using the default parameters: gap opening penalty, 10; gap extension penalty, 0.1; and delay divergent cutoff, 25%, and evolutionary distances were computed using MEGA5.10 with the Poisson correction method. For the phylogenetic analysis, a neighbor-joining tree was constructed using MEGA5.0. Bootstrap values obtained after 1000 replications are indicated on the branches. The scale represents 0.1 amino acid substitutions per site.

## Results and Discussion

### Illumina Sequencing and *de novo* Assembly

In order to find more genes involved in the biosynthesis of scutellarin and chlorogenic acids, total RNA was extracted from the fully-expanded young fresh leaves and the flowers at different stages. Leaves and flowers are the primary medicinal parts of *E. breviscapus*. RNA samples from different tissues were mixed in equal proportions and then used for mRNA preparation, fragmentation, and cDNA synthesis. The cDNA was sequenced using the Illumina Genome Analyzer IIx platform and the resulting sequencing data were subjected to bioinformatic analysis. The paired-end sequencing yielded 2×100 bp independent reads from either end of the cDNA fragment. In this study, 64,605,972 raw reads were generated from a 200 bp insert library. After trimming adaptor sequences and removing the low quality reads (Q20<20), 62,595,942 clean reads were obtained. Among these clean reads, 93.04% reads were deemed high quality reads, and were selected for further analysis.

Reference genome sequence was unavailable for *E. breviscapus*, thus all the clean reads (62,595,942) were assembled *de novo* using Trinity, with optimized k-mer length of 25; all short sequences were assembled into 73,092 unigenes within a length range from 201 to 12,242 bp, comprising a total length of 66,962,717 bp. The average length of all unigenes is 916 bp, and there was 23,844 unigenes (32.62%) longer than 1000 bp. An overview of the sequencing and assembly is outlined in Table 1.

**Table 1:** Summary of Illumina Paired-end sequencing and assembly for *E. breviscapus*.

Database	Number	Total Length (bp)
Total Clean reads	62,595,942	625,959,420
Q20 percentage	93.04%	
Number of contigs	81,406	76,760,306
Average length of contigs	943	
Max length of contigs	12,242	
Min length of contigs	201	
Contig size N50	1,452	
Number of unigenes	73,092	66,962,717
Average length of unigenes	916	
Max length of unigenes	12,242	
Min length of unigenes	201	
Unigene size N50	1,434	

**Figure 2:**
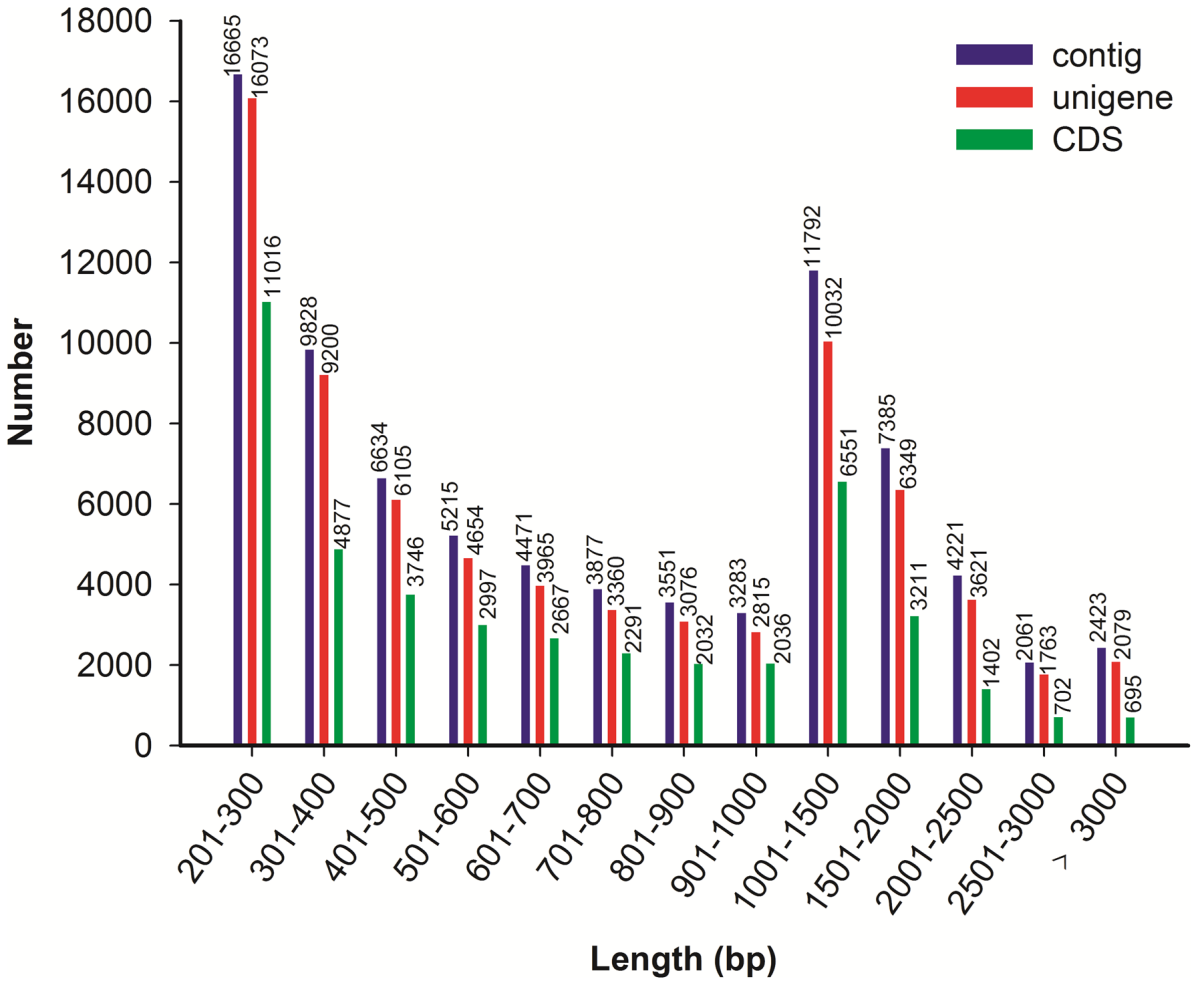
Overview of *E. breviscapus* transcriptome assembly.

The CDS (coding DNA sequence) in all unigenes were also detected by comparing the sequences to protein databases Nr, SwissProt, KEGG, and COG using BLSATX (E-value <10^−5^). For the unigene that do not have similarity in all protein databases, ESTScan was used for detecting CDS [50]. Most unigenes (44,223, 60.50%) were identified to have CDS, among them, 33.01% unigenes have CDS longer than 100 bp. The length distributions of the contigs, unigenes, and CDS were shown in Figure 2.

The Illumina platform has been widely used for *de novo* transcriptome sequencing in many organisms. RNA-seq does not require a reference genome to gain useful transcriptomic information. This makes RNA-Seq technology particularly useful for non-model organisms that often lack genomic sequence data [51]. In this study, these results indicated again that Illumina paired-end sequencing technology is useful for the *de novo* sequencing and assembly of transcriptome of non-model plant species. As far as we know, this is the first report on a large scale of transcriptome sequencing and analysis in *E. breviscapus*. These data will contribute significantly to further genome-wide research and analyses in this herb.

### Functional annotation

**Figure 3:**
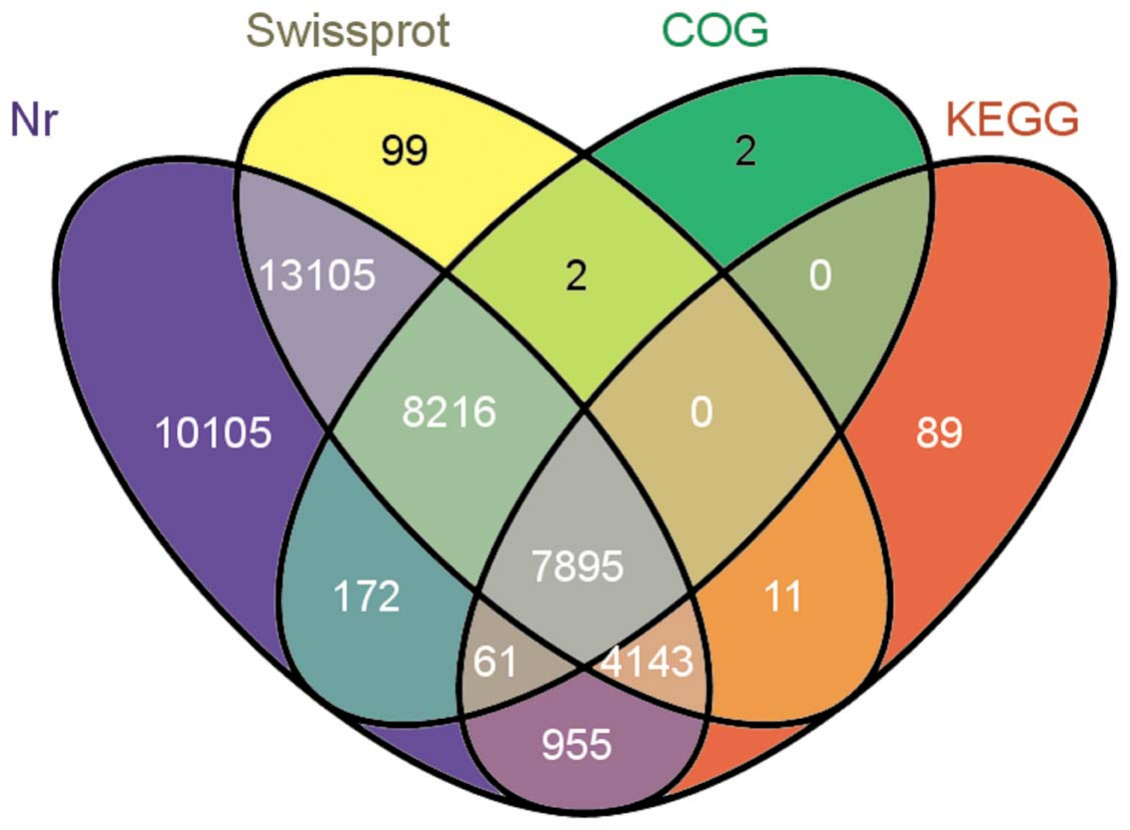
The number of unigenes annotated by BLASTX with an E-value threshold of 10^−5^ against protein databases. The numbers in the circles indicate the number of unigenes annotated by single or multiple databases.

The unigenes were aligned to the four public protein databases (Nr, Swiss-Prot, KEGG, and COG). A total 44,855 unigenes (61.37%) were annotated in the public databases (Table 2). Of these, 7,895 unigenes were common through the four databases, 10,105 unigenes annotated uniquely in Nr databases, 99 uniquely in Swiss-Prot databases, 2 unigenes in COG databases, and 89 unigenes uniquely in KEGG databases (Figure 3). Furthermore, most of the identified unigenes showed a high similarity to those from *Vitis vinifera* (10,543, 28.62%), *Arabidopsis thaliana* (4,559, 12.38%), *Glycine max* (4,444, 12.07%), *and Medicago truncatula* (4,222, 11.46%) (File S3). This suggests the *E. breviscapus* genome is more closely related to *V. vinifera* genome than to other sequenced plant genomes.

**Table 2:** Summary of the annotation percentage of *E. breviscapus* unigenes as compared to public databases.

Database	Number of unigenes	Annotation percentage (%)
Nr	44,652	61.09
SwissProt	33,471	45.79
KEGG	13,154	18.00
COG	16,348	22.37
All	44,855	61.37
Total	73,092	

We also compared the length of unigenes with the percentage which matches against the sequences in Nr and Swiss-Prot database, and found that the longer unigenes had the higher percentage, especially those longer than 1000 bp, but the unigenes of the length of 600 to 1000 bp had a lower percentage of their matches (File S4). For E-value and similarity distributions in the Nr database, 52.63% unigenes showed a significant similarity (E-value<1e^−50^) and 14.28% unigenes had a high similarity of greater than 80%. In the Swiss-Prot database, the percentages were 44.37% and 9.97%, respectively (File S5).

### Gene Ontology Classification

**Figure 4:**
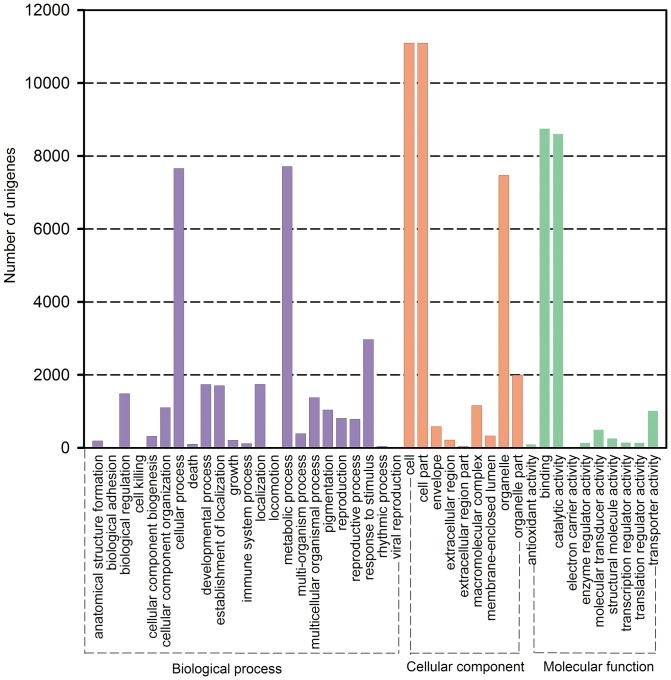
Gene Ontology classification of assembled unigenes. Total 17,156 unigenes were categorized into three main categories: biological process, cellular component and molecular function.

GO assignments were used to classify the functions of all unigenes. Based on sequence similarity, 17,156 unigenes were assigned to one or more ontologies. Totally, 31,527 unigenes were grouped under biological processes, 33,986 unigenes under cellular components, 19,671 unigenes under molecular functions. Binding (8,754 unigenes, 44.50%) and catalytic activity (8,606 unigenes, 43.75%) were the most highly represented groups under the molecular function category. For the biological process class, the assignments were mainly given to the metabolic process (7,714 unigenes, 24.47%) and cellular process (7660 unigenes, 24.30%) (Figure 4; File S6). These GO annotations provide a valuable clue for investigating the specific processes, molecular functions, and cellular structures of *E. breviscapus* transcriptome.

### Functional Classification by COG

**Figure 5:**
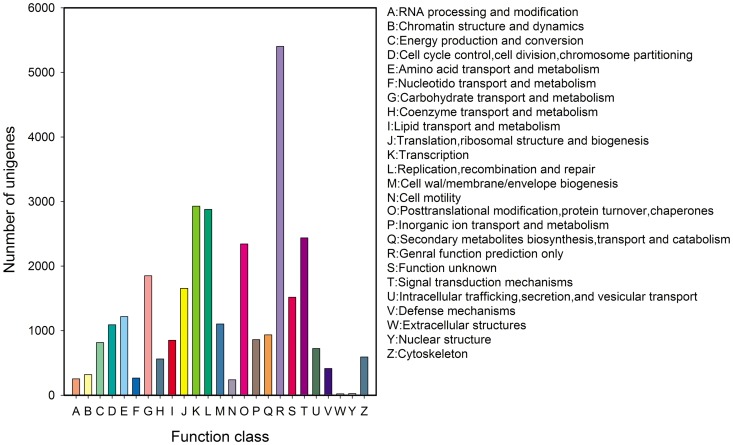
COG function classification of *E. breviscapus* unigenes.

All unigenes were subjected to a search against the COG database for functional prediction and classification. In total, 31,305 unigenes were annotated and grouped into 25 COG classifications. The largest cluster was for general function prediction only (5,403, 17.26%), followed by transcription (2,927, 9.35%), replication, recombination and repair (2,878, 9.20%), signal transduction mechanisms (2,438, 7.79%), posttranslational modification, protein turnover, and chaperones (2,343, 7.48%), carbohydrate transport and metabolism (1,849, 5.90%), and translation, ribosomal structure and biogenesis (1,655, 5.29%) (Figure 5).

### Functional Classification by KEGG

**Figure 6:**
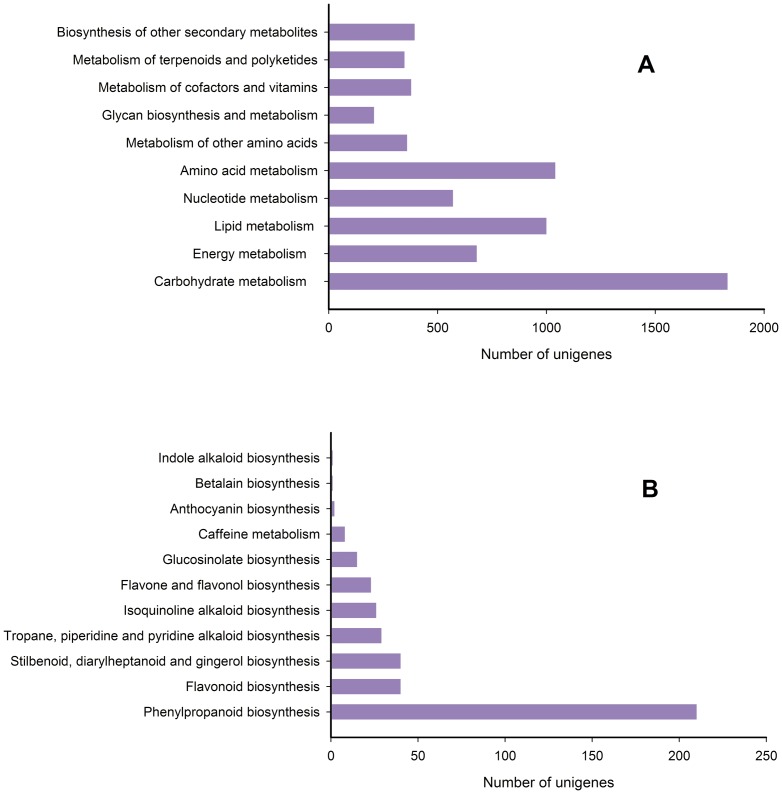
Pathway assignment based on the KEGG. (A) Classification based on metabolism categories; (B) Classification based on biosynthesis of other secondary metabolites.

To further identify the active biochemical pathways in *E. breviscapus*, we mapped the unigenes to the reference canonical pathways in the Kyoto Encyclopedia of Genes and Genomes (KEGG). Pathway-based analysis can help us understand the biological functions of genes. Firstly, based on a comparison against the KEGG database using BLASTx with an E-value cut off of <10^−5^, of the 73,092 unigenes, 13,154 unigenes (18.00%) had significant matches in the database and were assigned to 125 KEGG pathways. Among them, about 6,814 unigenes were assigned to metabolic pathways (File S7). Metabolic pathways had the largest number of unigenes (3,234 members, 18.49%), followed by secondary metabolite biosynthesis (1,543 members, 8.82%), RNA transport (438 members, 2.50%), protein processing in endoplasmic reticulum (428 members, 2.45%), and spliceosome (426 members, 2.43%). 6,814 unigenes were assigned to metabolic pathways. As shown in Figure 6A, 1,832 unigenes were clustered into carbohydrate metabolism, followed by amino acid metabolism(1,041 unigenes), lipid metabolism (1,000 unigenes), energy metabolism (680 unigenes), nucleotide metabolism (571 unigenes), biosynthesis of others secondary metabolites (395 unigenes), metabolism of cofactors and vitaminsand (379 unigenes), metabolism of other amino acids (360 unigenes), metabolism of terpenoids and polyketides (348 unigenes), and glycan biosynthesis and metabolism (208 unigenes).

In the other secondary metabolites, 11 subcategories comprised 395 unigenes, the most represented categories of which were phenylpropanoid biosynthesis (210 members, 1.20%), flavonoid biosynthesis (40 members, 0.23%), stilbenoid, diarylheptanoid, and gingerol biosynthesis (40 members, 0.23%), tropane, piperidine, and pyridine alkaloid biosynthesis (29 members, 0.17%), isoquinoline alkaloid biosynthesis (26 members, 0.15%), flavone and flavonol biosynthesis (23 members, 0.13%), glucosinolate biosynthesis (15 members, 0.09%), caffeine metabolism (8 members, 0.05%), anthocyanin biosynthesis (2 members, 0.01%), betalain biosynthesis (1 members, 0.005%), and indole alkaloid biosynthesis (1 members, 0.005%) (Figure 6B). In addition to metabolism pathways, genetic information processing genes (4,175) and cellular processes genes (693) were highly represented categories.

### Candidate genes encoding enzymes involved in scutellarin biosynthesis

Based on the assignment of KEGG pathway, all of the genes encoding enzymes involved in flavonoid biosynthesis were found. The number of unigenes involved in the biosynthesis of secondary metabolites was shown in File S8. In particular, the transcripts encoding all the known enzymes involved in flavonoids (flavone and anthocyanin) biosynthesis were discovered in this Illumina dataset, including PAL (phenylalanine ammonia lyase), C4H (cinnamate 4-hydroxylase), 4CL (4-coumarate:CoA ligase), CHS (chalcone synthase), CHI (chalcone isomerase), FSII (flavone synthase II), F3H (flavanone 3-hydroxylase), F3'H (flavonoid 3′ hydroxylase), F3′5′H (flavonoid 3′5′ hydroxylase), DFR (dihydroflavonol 4-reductase), LDOX (leucoanthocyanidin dioxygenase), OMT (*O*-methyltransferase), UFGT (UDPG-flavonoid glucosyl transferase), and RT (rhamnosyl transferase) (Table 3; File S9).

**Table 3:** Transcripts involved in flavonoids and chlorogenic acids biosynthesis in *E. breviscapus*.

Gene name	EC number	unigene numbers
4CL, 4-coumarate: CoA ligase	6.2.1.12	44
C4H, cinnamate 4-hydroxylase	1.14.13.11	1
CHI, chalcone isomerase	5.5.1.6	8
CHS, chalcone synthase	2.3.1.74	11
DFR, dihydroflavonol 4-reductase	1.1.1.219	37
F3′5′H, flavonoid 3′5′ hydroxylase	1.14.13.88	1
F3′H, flavonoid 3′ hydroxylase	1.14.13.21	6
UFGT, UDPG-flavonoid glucosyl transferase	2.4.1.91	4
F3H, flavanone 3-hydroxylase	1.14.11.9	8
F7GAT, flavonoid 7-O-glucuronosyltransferases		8
FSII, flavone synthase II	1.14.13.87	1
HQT, hydroxycinnamoyl-CoA quinate hydroxycinnamoyl transferase	2.3.1.99	8
HCT, hydroxycinnamoyl-CoA shikimate/quinate hydroxycinnamoyl transferase	2.3.1.133	15
LDOX, leucoanthocyanidin dioxygenase	1.14.11.19	4
PAL, phenylalanine ammonia lyase	4.3.1.24	20
RT, rhamnosyl transferase	2.4.1.115	1
UGCT, UDP glucose: cinnamate glucosyl transferase	2.4.1.177	1
C3H, *p*-coumarate 3-hydroxylase	1.14.13.21	1

Considering that scutellarin is the major active component of *E. breviscapus*, there must be a flavonoid 6-hydroxylase (F6H), which converts apigenin to scutellarein, followed by glucuronidation catalyzing by flavonoid 7-*O*-glucuronosyltransferase (F7GAT). In plants, F6Hs have been insufficiently studied due to their restricted occurrence [52]. In *Chrysosplenium americanum*, flavonol 6-hydroxylase is a 2-oxoglutarate-dependent dioxygenase (ODD), which preferred methylated flavonols as substrates [13], but the F6H in soybean (*G. max*) is a cytochrome P450-dependent monooxygenase (CYP71D9), which catalyzed the conversion of flavanones more efficiently than flavones [14]. Recently, two flavone 6-hydroxylases were identified from *Ocimum basilicum* (CYP82D33) and *Mentha x piperita* (CYP82D62), both of which belong to a new CYP82 family, prefer 7-*O*-methylated derivative genkwanin as a substrate, and have very low 6-hydroxylase activity with apigenin. [15]. Therefore, F6H with activity of converting apigenin to scutellarein was not found.

**Figure 7:**
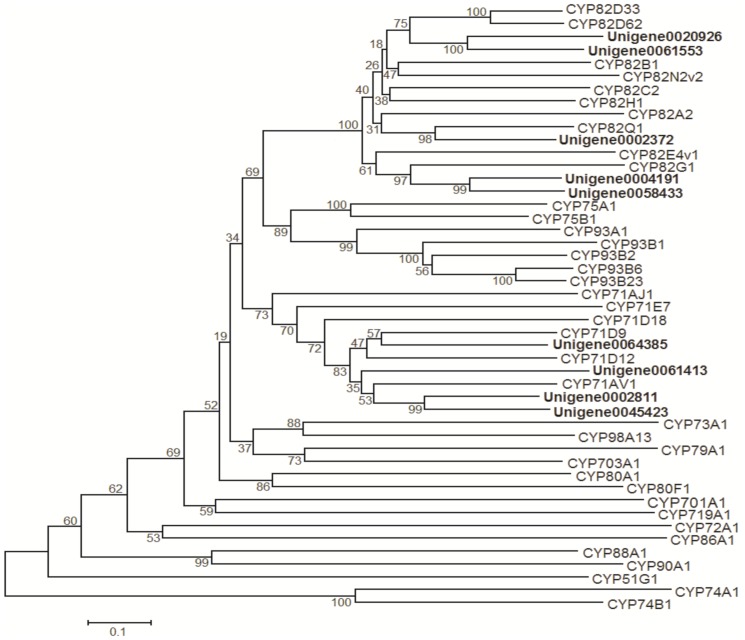
Phylogenetic tree of the *E. breviscapus* CYPs. Phylogenetic tree constructed based on the deduced amino acid sequences for the *E. breviscapus* CYPs (bold letters) and other plant CYPs. Protein sequences were retrieved from NCBI GenBank using the following accession numbers (source organism and proposed function, if any, are given in parentheses): CYP82D33, JX162212 (*Ocimum basilicum*, flavonoid 6-hydroxlase); CYP82D62, JX162214 (*Mentha x piperita*, flavonoid 6-hydroxylase); CYP82B1, AAC39454 (*Eschscholzia californica*, (S)-N-methylcoclaurine 3′-hydroxylase); CYP82N2v2, BAK20464 (*Eschscholzia californica*, protopine 6-hydroxylase); CYP82C2, O49394 (*A. thaliana*); CYP82H1, AAS90126 (*Ammi majus*); CYP82A2, CAA71515 (*Glycine max*); CYP82Q1, ABB20912 (*Stevia rebaudiana*); CYP82E4v1, ABA07805 (*Nicotiana tabacum*, nicotine demethylase); CYP82G1, NP_189154 (*Arabidopsis thaliana*, geranyllinalool catabolism); CYP75A1, CAA80266 (*Petunia x hybrid*, flavonoid 3′,5′-hydroxylase); CYP75B1, AAD56282 (*Petunia x hybrida*,flavonoid 3′-hydroxylase); CYP93A1, NP_001241186 (*G. max*, 3,9-dihydroxypterocarpan 6a-hydroxylase); CYP93B1, BAA22423 (*Glycyrrhiza echinata*, flavone synthase); CYP93B2, AAD39549 (*Gerbera hybrid*, flavone synthase); CYP93B6, BAB59004 (*Perilla frutescens var. crispa*, flavone synthase); CYP93B23, JX162213 (*Ocimum basilicum*, flavone synthase); CYP71AJ1, AAT06911 (*Ammi majus*, psoralen synthase); CYP71E7, AAP57704 (*Manihot esculenta*, cyanogenic glucoside oxim metabolism); CYP71D18, AAD44150 (*Mentha spicata*, limonene 6-hydroxylase), CYP71D9, CAA71514 (*G. max*, flavonoid 6-hydroxylase); CYP71D12, CAB56503 (*Catharanthus roseus*, tabersonine 16-hydroxylase); CYP71AV1, ABB82944 *(Artemisia annua*, amorpha-4,11-diene C-12 oxidase); CYP80A1, AAC48987 (*Berberis stolonifera*, berbamunine synthase); CYP80F1, ABD39696 (*Hyoscyamus niger*, littorine mutase); CYP73A1, CAA78982 (*Helianthus tuberosus*, trans-cinnamate 4-hydroxylase); CYP98A13, AAL99200 (*Ocimum basilicum*, p-coumaroyl shikimate 3′-hydroxylase); CYP79A1, AAA85440 (*Sorghum bicolor*, tyrosine *N*-hydroxylase); CYP703A1, BAA92894 (*Petunia x hybrid*, lauric acid hydroxylase); CYP701A1, AAG41776 (*Cucurbita maxima*, ent-kaurene oxidase); CYP719A1, BAB68769 (*Coptis japonica*, methylenedioxy bridgeforming enzyme); CYP72A1, AAA33106 (*C. roseus*, secologanin synthase); CYP86A1, P48422 (*A. thaliana*, fatty acid -hydroxylase); CYP88A1, AAC49067 (*Zea mays*, entkaurenoic acid oxidase); CYP90A1, Q42569 (*A. thaliana*, 6-oxocathasterone 23a-hydroxylase); CYP51G1, BAB61873 (*A. thaliana*, obtusifoliol 14-demethylase); CYP74A1, AAA03353 (*Linum usitatissimum*, allene oxide synthase); CYP74B1, AAA97465 (*Capsicum annuum*, fatty acid hydroperoxide lyase).

For discovering the candidate gene of F6H in *E. breviscapus*, the unigenes annotated to cytochrome P450 were collected and shown in File S10. Nine of them were annotated to CYP71 or CYP82 family, and the phylogenetic relationship between them and characterized CYP450s from other plants was depicted in Figure 7. We found that, one unigene (unigene0064385) is phylogenetically close to CYP71D9, and 2 unigenes (Unigene0020926 and Unigene0061553) are close to CYP82D33 and CYP82D62, thus these unigenes are regarded as candidates of F6H in *E. breviscapus*. The functional identification of these genes will be carried out in the future.

F7GAT transfers glucuronic acid from UDP-glucuronic acid (UDPGA) of sugar donor to the 7-OH group of flavonoids of sugar acceptor. F7GAT was firstly purified from *S. baicalensis*, which specifically uses UDPGA as the sugar donor and glucuronosylates the 7-OH group of flavones with *ortho*-substituents, such as scutellarein and baicalein [53]. F7GATs isolated from the plants within the Lamiales order are the members of the UGT88-related cluster [6]. In this study, we did not found any ortholog of F7GAT in the transcriptome dataset of *E. breviscapus*. The reason is not clear.

### Transcripts encoding putative flavone-specific MYB transcription factors in *E. breviscapus*

**Figure 8:**
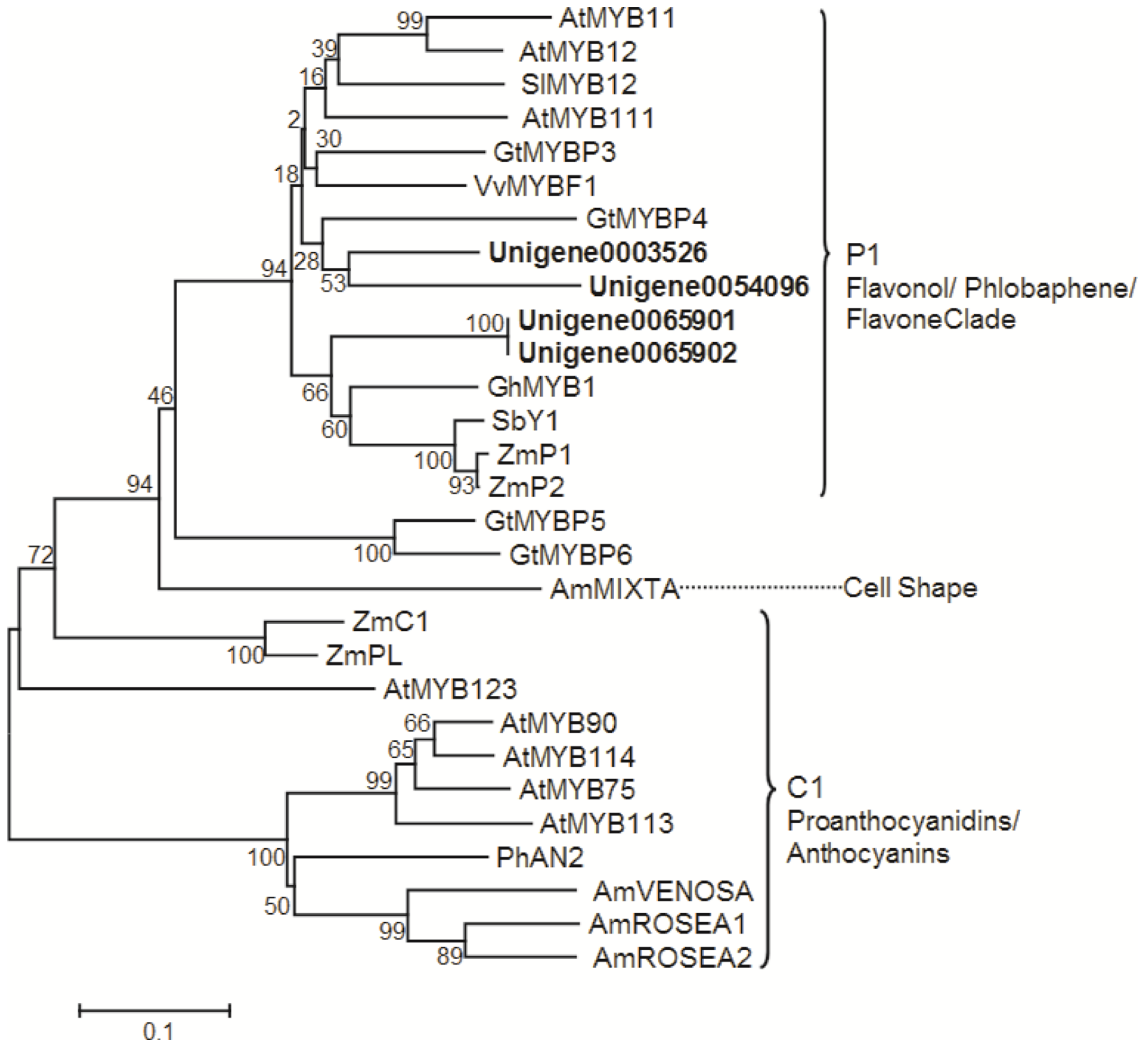
Phylogenetic tree of the *E. breviscapus* R2R3-MYB transcription factors. Phylogenetic tree constructed based on the deduced amino acid sequences for the *E. breviscapus* R2R3-MYB transcription factors (bold letters) and other plant R2R3-MYB transcription factors. Accession numbers in the NCBI GenBank database are as follows: AtMYB111, *Arabidopsis thaliana* (NM124310); GtMYBP3, *Gentiana triflora* (AB733016); VvMYBF1, *Vitis vinifera* (FJ948477); SlMYB12, *Solanum lycopersicum* (EU419748); AtMYB11, *Arabidopsis thaliana* (NM116126); AtMYB12, *Arabidopsis thaliana* (NM130314); GtMYBP4, *Gentiana triflora* (AB289446); GhMYB1, *Gerbera hybrid* (AJ554697); SbY1, *Sorghum bicolor* (AY860968); ZmP1, *Zea mays* (M73028); ZmP2, Zea mays (AF210616); ZmC1, *Zea mays* (MZEMYBAA); ZmPL, *Zea mays* (AF015268); AtMYB123, *Arabidopsis thaliana* (AJ299452); AtMYB75, *Arabidopsis thaliana* (AF325123); AtMYB114, *Arabidopsis thaliana* (AY519567); AtMYB114, *Arabidopsis thaliana* (AY519567); AtMYB90, *Arabidopsis thaliana* (AF325124); GtMYBP5,*Gentiana triflora* (AB733616); GtMYBP6, *Gentiana triflora* (AB733617); AtMYB113, *Arabidopsis thaliana* (NP176811); PhAN2, *Petunia x hybrida* (AF146702); AmVENOSA, *Antirrhinum majus* (DQ275531); AmROSEA1, *Antirrhinum majus* (DQ275529); AmROSEA2, *Antirrhinum majus* (DQ275530); AmMIXTA, *Antirrhinum majus* (X79108).

Since scutellarin is a kind of flavone glucuronide derivative, so we focused on flavone-specific transcription factors (TFs), especially on R2R3-MYB. The R2R3-MYB TFs for anthocyanin and proanthocyanin belong to maize C1 homologues (R2R3-MYB), such as AN2 in petunia [54] and PAP1/PAP2 in *Arabidopsis* [55]. These R2R3-MYB TFs require interaction with basic helix–loop–helix (bHLH) and WD40 repeats (WDR) protein for regulating the structural genes expression in the late biosynthetic pathway. In contrast, the R2R3-MYB TFs regulating flavones, flavonols, and phlobaphene biosynthesis don't need to bind with other type of TFs. Such the R2R3-MYB TFs are the homologues of maize P1 (ZmP1), including AtMYB12, AtMYB11, and AtMYB111 in *Arabidopsis* [56], [57], VvMYBF1 in grapevine [58], and GtMYBP3 and GtMYBP4 in *Gentiana triflora* [59]. In the transcriptome dataset, a total of 846 unigenes were annotated to MYB TFs, among them, 4 unigenes are highly similar to AtMYB12 or AtMYB111 (File S11). Phylogenetic analysis showed that they all belonged to P1/subgroup 7 (Figure 8), suggesting that those might be the specific TFs for particularly regulate the genes involved in scutellarin biosynthesis. Characterizing the functions of those unigenes will help us deeply understand the regulatory mechanism of scutellarin biosynthesis.

### Genes encoding enzymes involved in chlorogenic acids biosynthesis

Three pathways have been proposed for the biosynthesis of CGA (Figure 1). Except HCGQT (hydroxycinnamoyl D-glucose: quinate hydroxycinnamoyl transferase), nearly all known enzymes involved in CGA biosynthesis had transcripts in this Illumina dataset, including UGCT (UDP glucose: cinnamate glucosyl transferase), C3H (*p*-coumarate 3-hydroxylase), HCT (hydroxycinnamoyl CoA shikimate/quinate hydroxycinnamoyl transferase), and HQT (hydroxycinnamoyl CoA quinate hydroxycinnamoyl transferase) (Table 3; File S9). Because of more transcripts encoding enzymes in pathway 2 and 3 (Figure 1), they are proposed to be the main biosynthesis pathways of CGA in *E. breviscapus*. The inhibiting of the expression of those genes by antisense RNA technology or RNA interference might reduce the contents of CGA and increase the content of scutellarin in this herb. Furthermore, the gene encoding enzyme which catalyzes CQA for the synthesis of di-CQAs is presumed to be the homologues of *HQT* gene, because they all transfer caffeoyl (Figure 1). In this study, we found that 8 transcripts were annotated to HQT (Table 3). Functional analysis of the unigenes will help us find the gene and enzyme that catalyzes the final step of di-CQA biosynthesis.

### EST-SSR Discovery: Distribution and Frequencies

Among molecular markers, SSRs are the most favored genetic markers because of their relative abundance, and they have been widely applied for molecular-assisted selection (MAS) in plant breeding programs [60]. All the unigenes obtained were searched in order to determine the frequency and distribution of SSRs. The potential SSRs were detected in all of the 73,092 unigenes using MISA software. A total of 11,077 SSRs were identified in 9,255 unigenes. Of all the SSR containing unigenes, 1,431 sequences contained more than one SSR and 848 SSRs were present in compound form (Table 4). The information of SSR derived from all unigene was shown in File S12. On average, we found 1.38 SSR per 10 Kb in this study, this is similar to the frequency in *Apium graveolens* (1 SSR per 10 KB) [61], but different from the frequencies in other plants [62], [63]. Among 11,077 SSRs identified, the tri-nucleotide repeat motifs were the most abundant types (45.19%), followed by di- (42.66%), tetra- (8.54%), hexa- (2.17%), and penta-nucleotide (1.44%) repeat motifs. SSRs with six tandem repeats (29.83%) were the most common, followed by five tandem repeats (29.05%), seven tandem repeats (15.02%), and four tandem repeats (10.03%) (Table 5). The most common type of di-nucleotide was AT/AT that accounted for 23.43% of the repeats, followed by AC/GT (11.95%) and AG/CT (7.28%). Among the tri-nucleotide repeats, both of AAT/ATT and ATC/ATG were the most frequent motifs (11.10%) (File S13). Using Primer3, total 20,865 pairs of primers were designed (File S14). These unique sequence-derived markers obtained in this study represent a valuable genetic resource for SSR mining and applications.

**Table 4:** Summary of SSR searching results.

Item	Number
Total number of sequences examined	73,092
Total size of examined sequences (bp)	66,962,717
Total number of identified SSRs	11,077
Number of SSR containing sequences	9,255
Avarage number of SSRs per 10 K	1.38
Number of sequences containing more than 1 SSR	1,431
Number of SSRs present in compound formation	848

**Table 5:** Distribution of identified SSRs using the MISA software.

Motif	Repeat numbers												Total	%
	4	5	6	7	8	9	10	11	12	13	14	≥15		
Di-	0	0	1,977	1,016	671	521	366	165	8	0	0	2	4,726	42.66
Tri-	0	3,029	1,291	643	40	0	0	0	1	2	0	0	5,006	45.19
Tetra-	752	163	30	1	0	0	0	0	0	0	0	0	946	8.54
Penta-	139	17	1	2	0	0	0	0	0	0	0	0	159	1.44
Hexa-	220	9	5	2	1	0	1	1	0	0	1	0	240	2.17
Total	1,111	3,218	3,304	1,664	712	521	367	166	9	2	1	2	11,077	100.00
%	10.03	29.05	29.83	15.02	6.43	4.70	3.31	1.50	0.08	0.02	0	0.02	100.00	-

### EST-SSR marker polymorphism

In this study, 36 primer pairs were randomly designed to validate the amplification and polymorphism in thirteen *E. breviscapus* accessions. 34 (94.40%) of the primer pairs successfully amplified fragments and 26 pairs (72.22%) produced PCR amplicons at the expected size. Among the successful primer pairs, 19 pairs (52.78%) exhibited polymorphisms among the thirteen *E. breviscapus* accessions (File S15). Polymorphism information content (PIC) values ranged from 0.13 to 0.50 with an average of 0.35.

**Figure 9:**
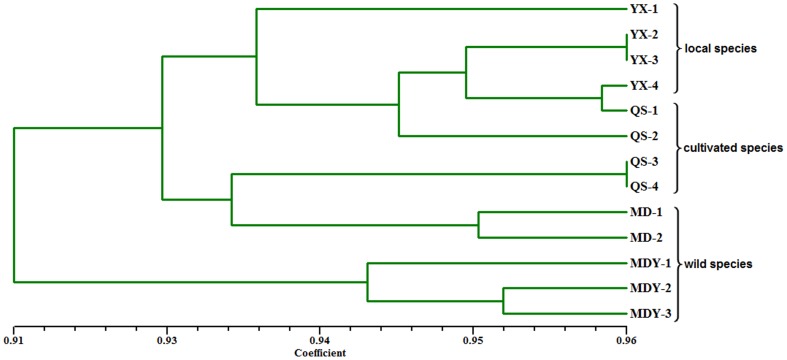
Dendrogram constructed with UPGMA clustering method among 13 different accessions of *E. breviscapus*.

The polymorphic SSR markers were then used to perform genetic correlation analysis among the 13 *E. breviscapus* accessions. The UPGMA clustering produced a dendrogram (Figure 9) that separated the 13 *E. breviscapus* accessions into three groups at the level of genetic similarity 0.93. All local species (YX-1,YX-2,YX-3,and YX-3) formed a cluster. Within the cultivated species, two lines (QS-3 and QS-4) formed a sub-cluster, and the other two lines (QS-1 and QS-2) did not cluster together with other cultivated species. Almost all wild species (MDY-1, MDY-2 and MDY-3) formed a cluster. It is noteworthy that two wild species (MD-1 and MD-2) formed a cluster with the other two cultivated species (QS-3 and QS-4), thus indicated that they may have a similar genetic background. These results demonstrate that SSRs can distinguish varieties without morphological diversities, and they will be a powerful tool for molecular breeding and genetics applications in this herb.

## Conclusions

*E. breviscapus* has been used as an herb for more than several centuries, but it was cultivated about only ten years. Thus, genomic information is urgently needed for molecular breeding. For a non-model plant which cannot be easily accessed for whole genome sequencing, the analyses of the transcripts provide an efficient and cost-effective way for genome exploring. This is the first attempt to elucidate the transcriptome of *E. breviscapus* using Illumina next-generation sequencing and *de novo* assembly. This study provided a large number of transcripts for gene function analysis and new gene discovery in this herb, especially for the candidate genes of flavone 6-hydroase and the transcription factors which might regulate scutellarin biosynthesis. The genes identified in this study will help to decipher the molecular mechanisms of secondary metabolism. This study will also contribute to the improvements on this species through marker-assisted breeding or genetic engineering.

## Supporting Information

Figure S1 ***Erigeron breviscapus*** **.** (A) Leaves and (B) flowers of *E. breviscapus*.
(TIF)

File S1 **Primer pairs validated in this study.**
(XLSX)

File S2 ***E. breviscapus*** **germplasms for polymorphism validation with EST-SSRs.**
(XLS)

File S3 **Top-hit species distribution for sequences from** ***E. breviscapus*** **submitted BLASTX against the NCBI-Nr database.**
(DOCX)

File S4 **Comparison of unigene length between hit and no hit unigenes.**
(DOCX)

File S5 **Characterization of searching the assembled unigenes against NCBI Nr and Swiss-Prot protein databases.**
(DOCX)

File S6 **Gene Ontology classification.**
(DOC)

File S7 **Mapping of** ***E. breviscapus*** **unigenes to KEGG biochemical pathways.**
(DOC)

File S8 **Number of unigenes which involved in the biosynthesis of secondary metabolites.**
(DOC)

File S9 **The main identified scutellarin and chlorogenic acids biosynthetic genes from** ***E. breviscapus*** **unigenes.**
(XLS)

File S10 **Cytochrome P450 discovery.**
(XLSX)

File S11 **MYB transcription factors in** ***E. breviscapus*** **unigenes.**
(XLSX)

File S12 **Information of SSR derived from all unigene.**
(XLSX)

File S13 **Frequency distribution of SSRs based on motif types.**
(DOCX)

File S14 **Sequences information SSR primers.**
(XLSX)

File S15 **Characteristics of 19 polymorphic EST-SSR primer pairs in 13** ***E. breviscapus*** **accessions.**
(DOCX)
