# Supplementary figures and images for: Analysis of the Transcriptome of Erigeron breviscapus Uncovers Putative Scutellarin and Chlorogenic Acids Biosynthetic Genes and Genetic Markers
Source: PLoS One. 2014 Jun 23;9(6):e100357. doi: 10.1371/journal.pone.0100357 (PMC4067309; doi:10.1371/journal.pone.0100357)

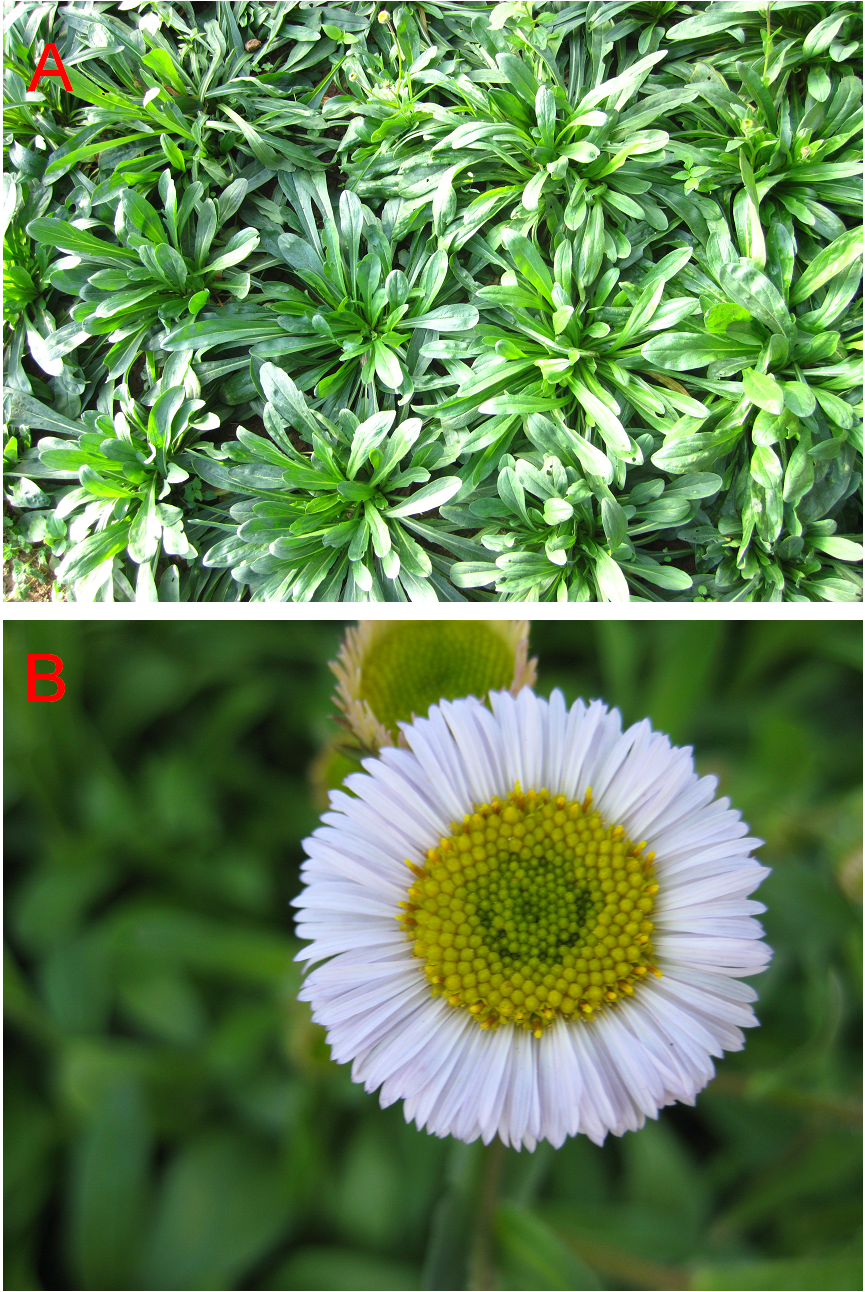

Supplement: Figure S1 — Erigeron breviscapus . (A) Leaves and (B) flowers of E. breviscapus. (TIF) [file pone.0100357.s001.tif]

**Figure 13.** Frequency distribution of SSRs based on motif types.


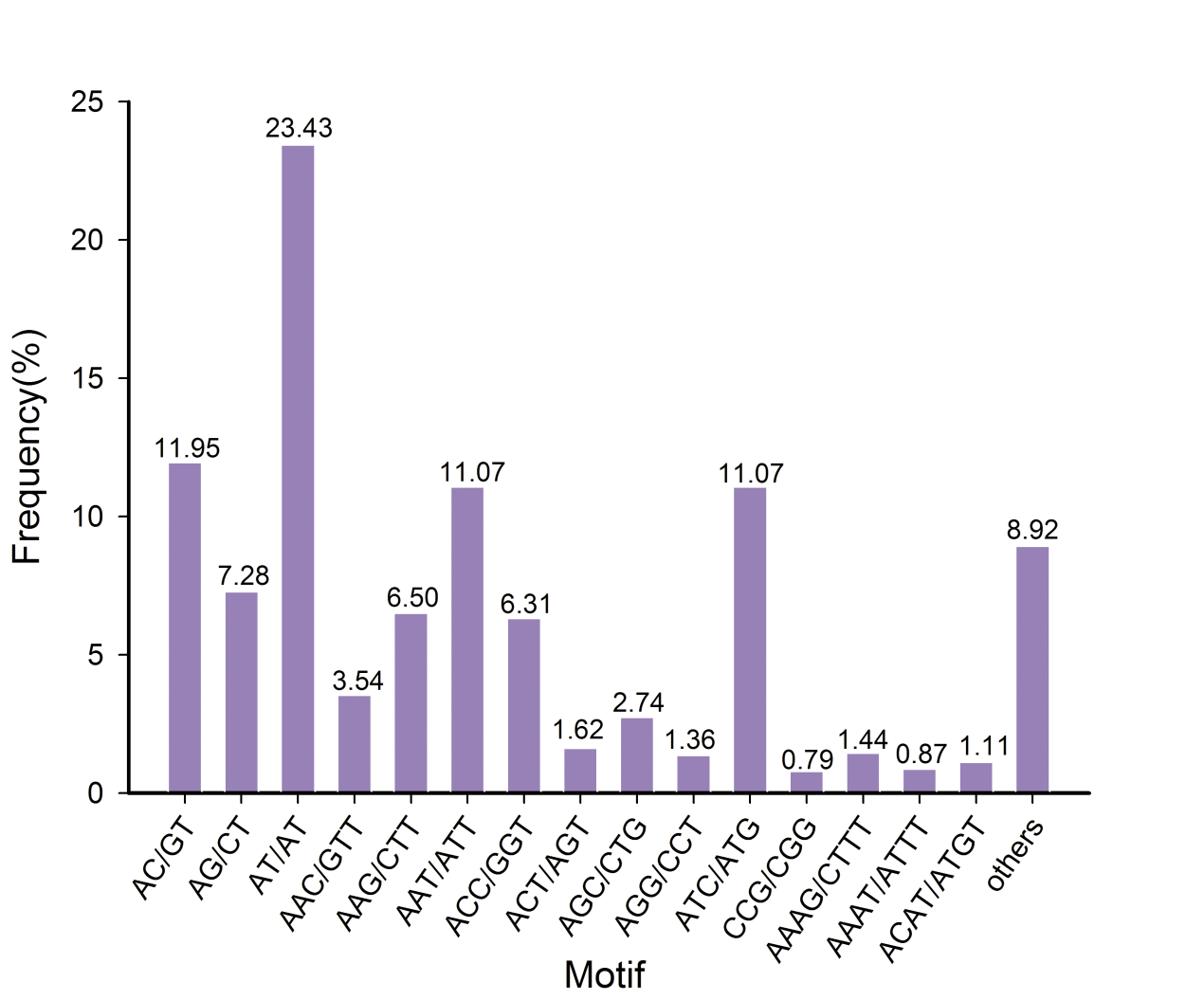

Supplement: File S13 — Frequency distribution of SSRs based on motif types. (DOCX) [file pone.0100357.s014.docx]
