# Supplementary material for: Analysis of the Transcriptome of Erigeron breviscapus Uncovers Putative Scutellarin and Chlorogenic Acids Biosynthetic Genes and Genetic Markers
Source: PLoS One. 2014 Jun 23;9(6):e100357. doi: 10.1371/journal.pone.0100357 (PMC4067309; doi:10.1371/journal.pone.0100357)

**File S3.** Top-hit species distribution for sequences from *E. breviscapus* submitted BLASTX against the NCBI-Nr database.


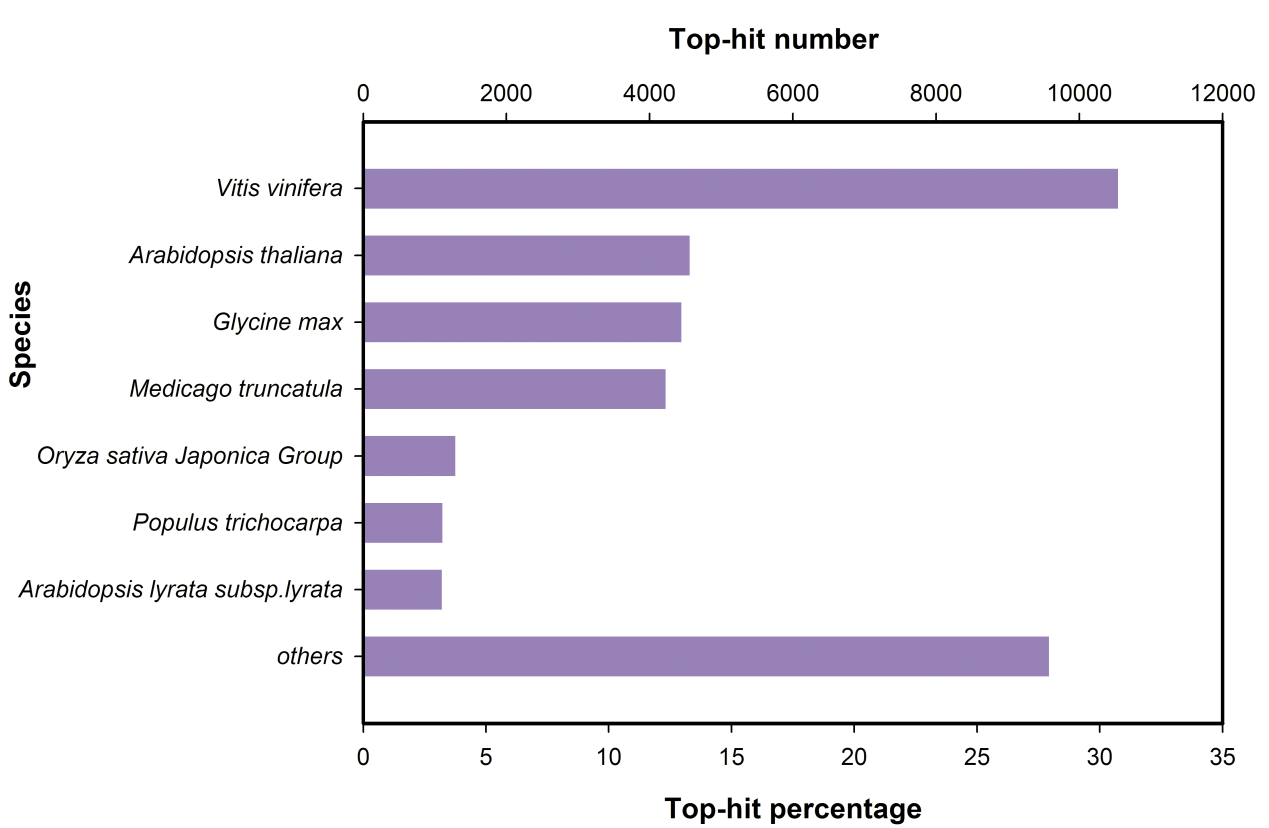

Supplement: File S3 — Top-hit species distribution for sequences from E. breviscapus submitted BLASTX against the NCBI-Nr database. (DOCX) [file pone.0100357.s004.docx]
