# Supplementary material for: Analysis of the Transcriptome of Erigeron breviscapus Uncovers Putative Scutellarin and Chlorogenic Acids Biosynthetic Genes and Genetic Markers
Source: PLoS One. 2014 Jun 23;9(6):e100357. doi: 10.1371/journal.pone.0100357 (PMC4067309; doi:10.1371/journal.pone.0100357)

**Figure S4. Comparison of unigene length between** **hit and no hit unigenes.** Longer unigenes were more likely to have BLAST matches in the NR database (A) and the Swiss-Prot database (B).


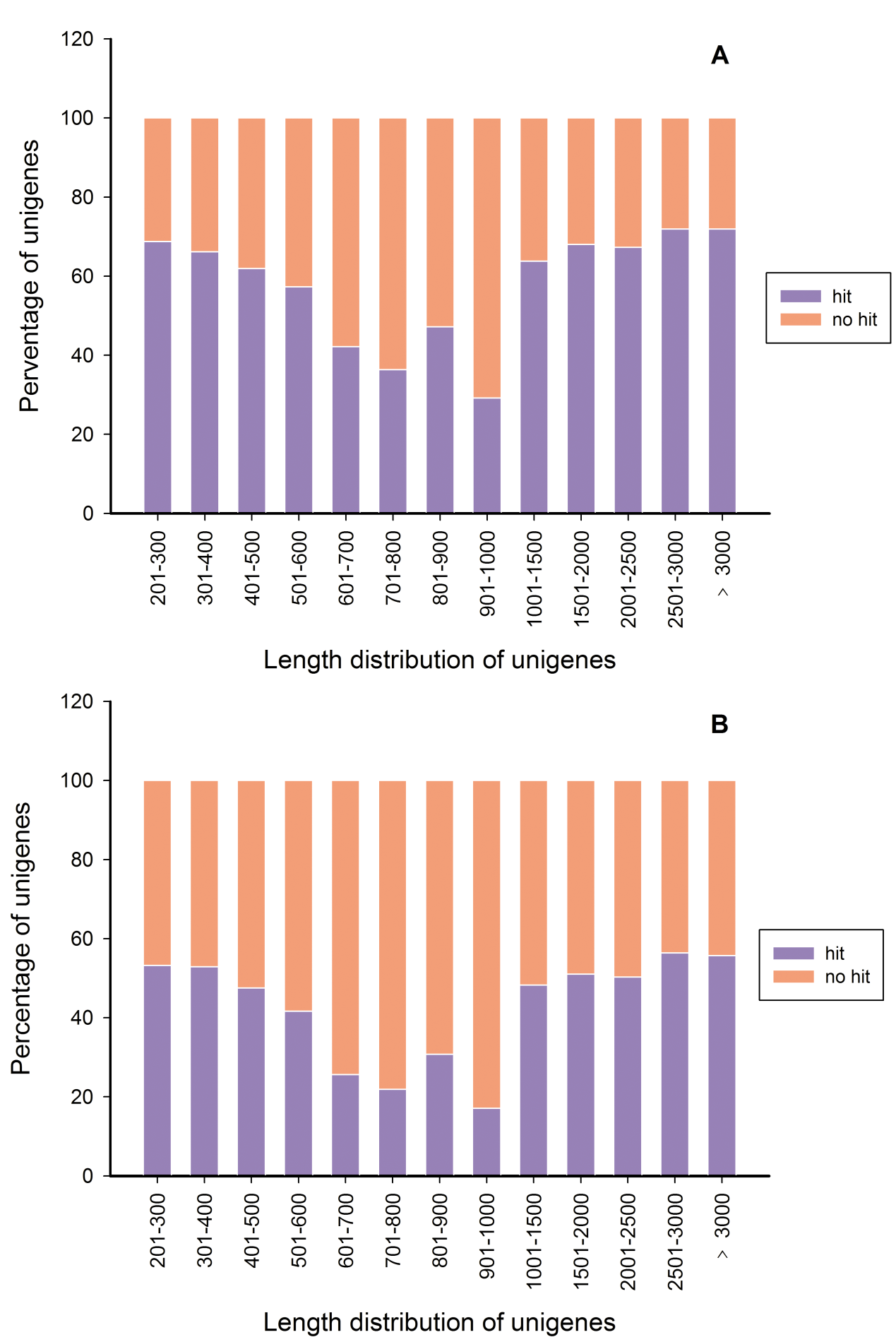

Supplement: File S4 — Comparison of unigene length between hit and no hit unigenes. (DOCX) [file pone.0100357.s005.docx]
