# Supplementary material for: Analysis of the Transcriptome of Erigeron breviscapus Uncovers Putative Scutellarin and Chlorogenic Acids Biosynthetic Genes and Genetic Markers
Source: PLoS One. 2014 Jun 23;9(6):e100357. doi: 10.1371/journal.pone.0100357 (PMC4067309; doi:10.1371/journal.pone.0100357)

**Figure S5. Characterization of searching the assembled unigenes against NCBI Nr and Swiss-Prot protein databases.** (A) E-value proportional frequency distribution of BLAST hits against the Nr database. (B) E-value proportional frequency distribution of BLAST hits against the Swiss-Prot database. (C) Similarity distribution of the top BLAST hits for the assembled unigenes with a cutoff of 1E-5 in Nr database. (D) Similarity distribution of the top BLAST hits for the assembled unigenes with a cutoff of 1E-5 in Swiss-Prot database.


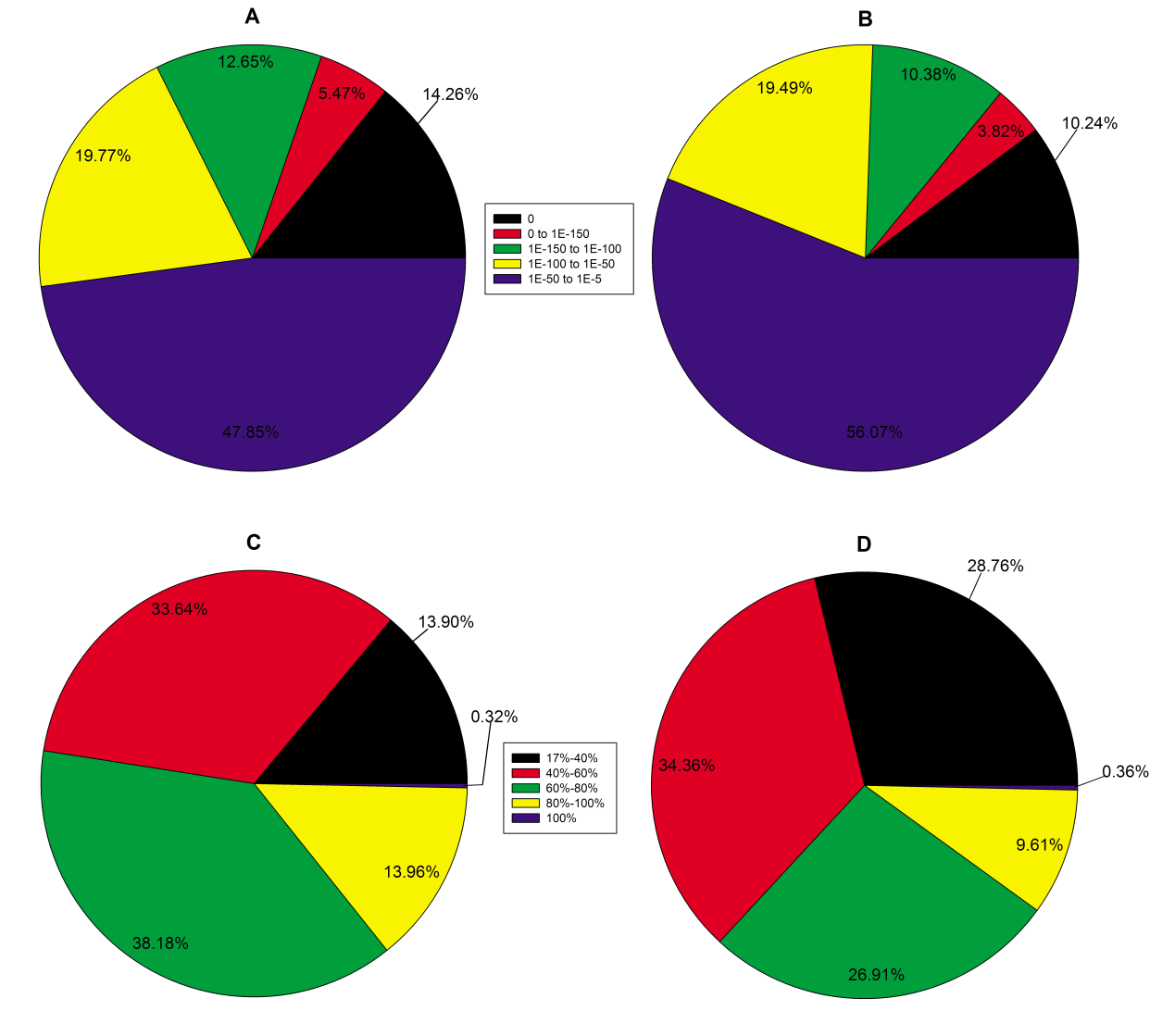

Supplement: File S5 — Characterization of searching the assembled unigenes against NCBI Nr and Swiss-Prot protein databases. (DOCX) [file pone.0100357.s006.docx]
