# Supplementary material for: Analysis of the Transcriptome of Erigeron breviscapus Uncovers Putative Scutellarin and Chlorogenic Acids Biosynthetic Genes and Genetic Markers
Source: PLoS One. 2014 Jun 23;9(6):e100357. doi: 10.1371/journal.pone.0100357 (PMC4067309; doi:10.1371/journal.pone.0100357)
Supplement: File S6 — Gene Ontology classification. (DOC) [file pone.0100357.s007.doc]

**Additional file 6. Gene Ontology classification**

| **Gene Ontology** | **Class** | **Number of Unigene (percentage)** |
| --- | --- | --- |
| **Biological process** | anatomical structure formation | 194 (0.23%) |
|  | biological adhesion | 3 (0.035%) |
|  | biological regulation | 1487 (1.75%) |
|  | cell killing | 4 (0.01%) |
|  | cellular component biogenesis | 318 (0.37%) |
|  | cellular component organization | 1105 (1.39%) |
|  | cellular process | 7660 (9.10%) |
|  | death | 102 (0.12%) |
|  | developmental process | 1737 (2.04%) |
|  | establishment of localization | 1705 (2.00%) |
|  | growth | 210 (0.25%) |
|  | immune system process | 121 (0.14%) |
|  | localization | 1742 (2.04%) |
|  | locomotion | 2 (0.032%) |
|  | metabolic process | 7714 (9.06%) |
|  | multi-organism process | 391 (0.46%) |
|  | multicellular organismal process | 1378 (1.63%) |
|  | pigmentation | 1037 (1.22%) |
|  | reproduction | 807 (0.95%) |
|  | reproductive process | 787 (0.92%) |
|  | response to stimulus | 2971 (3.49%) |
|  | rhythmic process | 44 (0.05%) |
|  | viral reproduction | 8 (0.001%) |
| **Cellular component** | cell | 11098 (13.03%) |
|  | cell part | 11098 (13.03%) |
|  | envelope | 584 (0.69%) |
|  | extracellular region | 211 (0.25%) |
|  | extracellular region part | 41 (0.05%) |
|  | macromolecular complex | 1161 (1.36%) |
|  | membrane-enclosed lumen | 326 (0.38%) |
|  | organelle | 7473 (8.77%) |
|  | organelle part | 1994 (2.34%) |
| **Molecular function** | antioxidant activity | 99 (0.12%) |
|  | binding | 8754 (10.28%) |
|  | catalytic activity | 8606 (10.10%) |
|  | electron carrier activity | 12 (0.01%) |
|  | enzyme regulator activity | 140 (0.16%) |
|  | molecular transducer activity | 499 (0.59%) |
|  | structural molecule activity | 259 (0.30%) |
|  | transcription regulator activity | 147 (0.17%) |
|  | translation regulator activity | 137 (0.16%) |
|  | transporter activity | 1018 (1.20%) |
| **Total** | **85184** (100%) | |
