# Supplementary material for: Analysis of the Transcriptome of Erigeron breviscapus Uncovers Putative Scutellarin and Chlorogenic Acids Biosynthetic Genes and Genetic Markers
Source: PLoS One. 2014 Jun 23;9(6):e100357. doi: 10.1371/journal.pone.0100357 (PMC4067309; doi:10.1371/journal.pone.0100357)
Supplement: File S7 — Mapping of E. breviscapus unigenes to KEGG biochemical pathways. (DOC) [file pone.0100357.s008.doc]

**Additional file 7. Mapping of *E. breviscapus* unigenes to KEGG biochemical pathways.**

| **KEGG categories represented** | **No. of uniques** |
| --- | --- |
| **Metabolism** | **6814** |
| Carbohydrate metabolism | 1832 |
| Energy metabolism | 680 |
| Lipid metabolism | 1000 |
| Nucleotide metabolism | 571 |
| Amino acid metabolism | 1041 |
| Metabolism of other amino acids | 360 |
| Glycan biosynthesis and metabolism | 208 |
| Metabolism of cofactors and vitamins | 379 |
| Metabolism of terpenoids and polyketides | 348 |
| Biosynthesis of other secondary metabolites | 395 |
| **Genetic Information Processing** | **4175** |
| Folding, sorting, and degradation | 1315 |
| Replication and repair | 669 |
| Translation | 1597 |
| Transcription | 594 |
| **Environmental Information Processing** | **497** |
| Membrane transport | 23 |
| Signal transduction | 99 |
| Plant hormone signal transduction | 375 |
| **Cellular Processes** | **693** |
| Transport and catabolism | 693 |
| **Organismal Systems** | **534** |
| Environmental adaptation | 65 |
| Immune system | 64 |
| Environmental adaptation | 60 |
| Plant-pathogen interaction | 345 |
| **Unassigned^a^** | **4777** |

^a^ Unassigned unique sequences are those that have significant similarities to known sequences in the KEGG database, but whose functions in biochemical pathways are unclear.
