# Supplementary material for: Analysis of the Transcriptome of Erigeron breviscapus Uncovers Putative Scutellarin and Chlorogenic Acids Biosynthetic Genes and Genetic Markers
Source: PLoS One. 2014 Jun 23;9(6):e100357. doi: 10.1371/journal.pone.0100357 (PMC4067309; doi:10.1371/journal.pone.0100357)
Supplement: File S8 — Number of unigenes which involved in the biosynthesis of secondary metabolites. (DOC) [file pone.0100357.s009.doc]

**Additional file 8. The number of unigenes which involved in the biosynthesis of secondary metabolites.**

| **Secondary metabolic pathways** | **No. of unigenes** |
| --- | --- |
| Phenylpropanoid biosynthesis | 210 |
| Flavonoid biosynthesis | 40 |
| Stilbenoid, diarylheptanoid and gingerol biosynthesis | 40 |
| Tropane, piperidine and pyridine alkaloid biosynthesis | 29 |
| Isoquinoline alkaloid biosynthesis | 26 |
| Flavone and flavonol biosynthesis | 23 |
| Glucosinolate biosynthesis | 15 |
| Caffeine metabolism | 8 |
| Anthocyanin biosynthesis | 2 |
| Betalain biosynthesis | 1 |
| Indole alkaloid biosynthesis | 1 |
| Terpenoid backbone biosynthesis | 117 |
| Carotenoid biosynthesis | 67 |
| Zeatin biosynthesis | 47 |
| Limonene and pinene degradation | 39 |
| Diterpenoid biosynthesis | 36 |
| Monoterpenoid biosynthesis | 16 |
| Brassinosteroid biosynthesis | 16 |
| Sesquiterpenoid biosynthesis | 10 |
| Citrate cycle (TCA cycle) | 132 |
| Amino sugar and nucleotide sugar metabolism | 177 |
| Pentose phosphate pathway | 81 |
| Ascorbate and aldarate metabolism | 58 |
| Glycolysis / Gluconeogenesis | 265 |
| Starch and sucrose metabolism | 326 |
| Glyoxylate and dicarboxylate metabolism | 96 |
| Steroid biosynthesis | 52 |
| Pantothenate and CoA biosynthesis | 57 |
| Porphyrin and chlorophyll metabolism | 85 |
| Ubiquinone and other terpenoid-quinone biosynthesis | 81 |
| Phenylalanine, tyrosine and tryptophan biosynthesis | 62 |
| Glycine, serine and threonine metabolism | 110 |
| Cysteine and methionine metabolism | 122 |
| Valine, leucine and isoleucine degradation | 103 |
| Valine, leucine and isoleucine biosynthesis | 44 |
| Alanine, aspartate and glutamate metabolism | 77 |
| Lysine biosynthesis | 23 |
| Histidine metabolism | 39 |
| Cyanoamino acid metabolism | 98 |
| Arginine and proline metabolism | 115 |
