# Supplementary material for: Analysis of the Transcriptome of Erigeron breviscapus Uncovers Putative Scutellarin and Chlorogenic Acids Biosynthetic Genes and Genetic Markers
Source: PLoS One. 2014 Jun 23;9(6):e100357. doi: 10.1371/journal.pone.0100357 (PMC4067309; doi:10.1371/journal.pone.0100357)
Supplement: File S15 — Characteristics of 19 polymorphic EST-SSR primer pairs in 13 E. breviscapus accessions. (DOCX) [file pone.0100357.s016.docx]

**File S15.** Characteristics of 19 polymorphic EST-SSR primer pairs in 13 *E. breviscapus* accessions.

| **Primer** | **Forward primer (5’-3’)** | **Reverse primer (5’-3’)** | **SSRs** | ***No*** | ***Ne*** | ***I*** | **NP** | **PPB** | **PIC** |
| --- | --- | --- | --- | --- | --- | --- | --- | --- | --- |
| P001 | ATGGAGCGGCAGTTACTATAATCTT | TCTTCAACTGATCTACGTGTCAAAG | (ACA)6 | 1.28 | 1.21 | 0.17 | 2.5 | 27.78 | 0.36 |
| P004 | GAGAGATCGATCGAGAAAGTAAATG | CGGTGGTGGTTTAGGCTCAG | (CAA)7 | 1.22 | 1.14 | 0.13 | 2.0 | 22.22 | 0.47 |
| P010 | CCGCTTCCCTTTGTAATTAACATTC | CAGAGAGATTTGTACTTGCGAAGAT | (TA)10 | 1.42 | 1.29 | 0.25 | 3.8 | 41.67 | 0.48 |
| P019 | AGACGACCGTAGATCAAATAAGATG | GTAATCAGCAGCACAATCGAAATG | (A)10 | 1.31 | 1.21 | 0.18 | 2.8 | 30.55 | 0.40 |
| P023 | CCTCGATAATATGTGTTTGGTGAAG | GTTGATCAGGAGATCATCATCATTG | (CAC)7 | 1.19 | 1.12 | 0.11 | 1.8 | 19.44 | 0.45 |
| P028 | TTTACTCTTCTCCACCTTCCTCTAC | CCGTTCTTCTTTGCCTTAATTC | (TAA)6 | 1.25 | 1.19 | 0.16 | 2.3 | 25.00 | 0.16 |
| P049 | CCCATATTCCTTGATCGAATTCTTCTG | ATACGAAACCAGGAGTCGTTATTGT | (CCA)7 | 1.11 | 1.08 | 0.07 | 1.0 | 11.11 | 0.25 |
| P052 | ATTGATCTAATGTGGCGGTGAT | CTGAAGTAAACTCCAGCAACAACTT | (ATC)7 | 1.08 | 1.05 | 0.19 | 0.8 | 8.33 | 0.23 |
| P053 | CACCGGAGCACTACGAATC | CACTCTTCTCACCACCACTAATTCT | (GTG)6 | 1.25 | 1.18 | 0.15 | 2.3 | 25.00 | 0.50 |
| P065 | GCCAAATCCACTATCCGAATTT | CTTGCATAGCAAAGATCCCAGTAT | (A)10 | 1.33 | 1.27 | 0.21 | 3.0 | 33.33 | 0.33 |
| P068 | AGCACCCAACACCAATCAAAT | GTTGAGACCAGGAACAAGTATGATG | (CAT)7 | 1.19 | 1.12 | 0.11 | 1.8 | 19.44 | 0.30 |
| P087 | ATTTGCGAGCCTTCTCCAATTT | CAAAGATGAGAGAATTATCCGACAC | (TCA)6 | 1.22 | 1.17 | 0.14 | 2.0 | 22.22 | 0.45 |
| P090 | GTGGTGTGTGGTGTCTTCTATGAT | CTCTCTGTTAATTCCCAACAACAAG | (GGT)6 | 1.25 | 1.17 | 0.15 | 2.3 | 25.00 | 0.50 |
| P095 | CGGAAACATGCTGCTCTTATTT | AAATGGTCCAGTTGAAAGCTC | (T)11 | 1.08 | 1.05 | 0.05 | 0.8 | 8.33 | 0.27 |
| P099 | GAGGTTCACTTGATTGTTGTTCAG | ATGTATCATATCAGATGGGAAGGTG | (ATC)6 | 1.08 | 1.05 | 0.05 | 0.8 | 8.33 | 0.35 |
| P106 | CTTTATCAATCGTCAGCTTCTCACT | CGCAACACTACCAGATTCTATTCAT | (CTCCAT)5 | 1.25 | 1.16 | 0.14 | 2.3 | 25.00 | 0.13 |
| P107 | ATGGTTTACGCATCATCAGATAGAC | CAACCAATGCCTACTTAACTTAACC | (CA)10 | 1.14 | 1.08 | 0.08 | 1.3 | 13.89 | 0.41 |
| P108 | CATACTCATGTCACTACCAAATCAGAC | GTGTTTGGATGCCAATTGAT | (T)10 | 1.25 | 1.17 | 0.15 | 2.3 | 25.00 | 0.29 |
| P109 | ATATGTATGTATATGGCACGAGGAC | GTCATATAACCACCGTCATAACCAT | (TA)11 | 1.33 | 1.21 | 0.19 | 3.0 | 33.33 | 0.37 |
| Mean | | |  | 1.22 | 1.15 | 0.14 | 2.0 | 22.37 | 0.35 |

*No*: Observed number of alleles; *Ne*: Expective number of alleles; *I*: Shannon’s information index; NP: Number of polymorphic loci; PPB: Percentage of polymorphic loci; PIC: Polymorphism Information Content.
